# RESULTS OF MECHANIC VERSUS MOTORIZED STAPLER USED IN GASTRIC SURGERY: PROSPECTIVE STUDY

**DOI:** 10.1590/0102-6720202400025e1818

**Published:** 2024-08-30

**Authors:** Italo BRAGHETTO, Gustavo CZWIKLITZER, Owen KORN, Percy BRANTE, Ana BURGOS

**Affiliations:** 1 Digestive and Bariatric Surgical Unit, Redsalud Providencia, Santiago, Chile.

**Keywords:** Gastrectomy, Gastric Bypass, Sutures, Surgical Staplers, Gastrectomia, Derivação Gástrica, Suturas, Grampeadores Cirúrgicos

## Abstract

**BACKGROUND:**

Mechanic sutures represent an enormous benefit for digestive surgery in decreasing postoperative complications. Currently, the advantages of motorized stapler are under evaluation.

**AIMS:**

To compare the efficacy of mechanic versus motorized stapler in gastric surgery, analyzing rate of leaks, bleeding, time of stapling, and postoperative complications.

**METHODS:**

Ninety-eight patients were submitted to gastric surgery, divided into three groups: laparoscopic sleeve gastrectomy (LSG) (n=47), Roux-en-Y gastric bypass (LRYGB) (n=30), and laparoscopic distal gastrectomy (LDG) (n=21). Motorized staplers were employed in 61 patients. The number of firings, number of clips, time of total firings, total time to complete the surgery, and postoperative outcome were recorded in a specific protocol.

**RESULTS:**

Patients submitted to LSG, LRYGB, and LDG recorded a shorter time to complete the procedure and a smaller number of firings were observed using motorized stapler (p<0.0001). No differences were identified regarding the number of clips used in patients submitted to LSG. In the group that used mechanic stapler to complete gastrojejunostomy, jejuno-jejuno-anastomosis, and jejunal transection, it was observed more prolonged time of firing and total time for finishing the procedure (p=0.0001). No intraoperative complications were found comparing the two devices used. Very similar findings were noted in the group of patients undergoing LDG.

**CONCLUSIONS:**

The motorized stapler offers safety and efficacy as demonstrated in prior reports and is relevant since less total time of surgical procedure without intraoperative or postoperative complications were confirmed.

## RESUMO

**RACIONAL:**

As suturas mecânicas representam um enorme benefício para a cirurgia digestiva na redução de complicações pós-operatórias. Atualmente, as vantagens do grampeador motorizado estão em avaliação.

**OBJETIVOS:**

Comparar a eficácia do grampeador mecânico *versus* motorizado em cirurgia gástrica, analisando a porcentagem de fístulas, sangramento, tempo de grampeamento e complicações pós-operatórias.

**MÉTODOS:**

Noventa e oito pacientes foram submetidos à cirurgia gástrica, divididos em três grupos: gastrectomia vertical laparoscópica (GVL) (n=47), bypass gástrico em Y-de-Roux (BPGY) (n=30) e gastrectomia distal laparoscópica (GDL) (n=21). Foram empregados grampeadores motorizados em 61 pacientes. O número de disparos, número de clipes, tempo total de disparos, tempo total de finalização da cirurgia e resultados pós-operatórios foram registrados em protocolo específico.

**RESULTADOS:**

Os pacientes submetidos a GVL, BPGY e GDL registraram menor tempo para finalização do procedimento e foi observado menor número de disparos com grampeador motorizado (p<0,0001). Não foram identificadas diferenças quanto ao número de clipes utilizados nos pacientes submetidos à GVL. No grupo que utilizou grampeador mecânico, para realização de gastrojejunostomia, jejuno-jejuno-anastomose e transecção jejunal, foi observado maior tempo de disparo e tempo total para finalização do procedimento (p=0,0001). Não foram encontradas complicações intraoperatórias comparando os dois dispositivos utilizados. Achados muito semelhantes foram notados no grupo de pacientes submetidos à GDL.

**CONCLUSÕES:**

O grampeador motorizado oferece segurança e eficácia, conforme demonstrado em estudos anteriores, sendo relevante, uma vez que foi confirmado menor tempo total de procedimento cirúrgico, sem complicações intra ou pós-operatórias.

## INTRODUCTION

A high percentage of gastric surgical procedures are performed by laparoscopic approach for benign or malignant diseases and bariatric surgery. Today, surgery for early and advanced gastric cancer can be undertaken laparoscopically except for large tumors^19,21,36^. Currently, patients with gastrointestinal stromal tumors or long Barrett’s esophagus are also submitted to laparoscopic distal gastrectomy (LDG) compared to the 1980s and 1990s decades^8,25,29,31^. Since 1993 we have performed laparoscopic procedures for these patients^5,9,16^. Laparoscopic sleeve gastrectomy (LSG) or laparoscopic Roux-en-Y gastric bypass (LRYGB), the most frequent bariatric procedures worldwide, are conducted with the same approach^10,32^. The major improvement in the type of devices employed for this surgery allows for diminished postoperative complications like leaks or bleeding from stapled line sutures^3,7,12,18,24,30^.

In the past decades, leaks or bleeding after LSG ranged 0–8% and 0–3%, respectively, but these complications have been reduced significantly with the use of new stapler devices. (0.8% in LSG *versus* 1.6% in LRYGB)^6,12,14,15,34,41,47,48^.

The purpose of this prospective study was to compare the efficacy of mechanic stapler with the motorized device in performing gastric surgery regarding rate of leaks, bleeding, time of stapling, and postoperative complications.

## METHODS

### Patients

This is a prospective study including a total of 98 patients divided into three groups:

Group A: Patients submitted to LSG (n=47), divided into two subgroups. The first one using mechanic stapler (n=10) and the second using motorized Ezisurg^™^ stapler (n=37);

Group B: Patients submitted to LRYGB (n=30), divided into two subgroups. The first using a mechanic stapler (n=14) and the second one using a motorized Ezisurg^™^ stapler (n=16);

Group C: Patients submitted to LDG (n=21), operated on due to esophageal or gastric diseases (combined with fundoplication and hiatal hernia repair in 18 patients), also divided into two groups depending on the use of mechanic stapler (n=13) and the second one using motorized Ezisurg^™^ stapler (n=8).

Table 1 shows the demographic characteristics of each group.

**Table 1:** Use of mechanic or motorized stapler in 98 patients submitted to laparoscopic sleeve gastrectomy (n=47), laparoscopic Roux-en-Y gastric bypass (n=30), and laparoscopic distal gastrectomy (n=21): demographic characteristics.

	Mechanic n=37 Mean±SD	Motorized n=61 Mean±SD
Age	43±11.9	36±9.2
Group A	38±8.5	35±8.9
Group B	40.9±10.9	38.06±12.9
Group C	51.6±12.6	39.0±11.9
Sex		
Female (n)	27	53
Male (n)	10	7
BMI (kg/m^2^)	37±11.1	39.1±2.8
Group A	41.2±8.2	40.0±5.6
Group B	40.5±3.4	40.4±4.6
Group C	38.8±9.3	36.3±6.4

SD: standard deviation; BMI: body mass index.

The surgical procedures were performed by only two surgeons (IB and GC), employing laparoscopic procedures, using a mechanic stapler in 37 patients and an Ezisurg^™^ motorized stapler in 61 patients. The LSG was performed according to the technique described previously^24,25^. For gastric transection, either a mechanic stapler with a 60–3.5mms blue cartridge or an Ezisurg^™^ motorized stapler was used. Clips or stitches were used to stop excessive bleeding of the suture line after firing. In the LRYGB, the detailed technique described by Brazilian authors was adopted^26,27^. For gastric transection, mechanic stapler 60–3.5 mms blue cartridge and 45–2.5 mms blue cartridge were employed only in a few cases for finishing the transection of the gastric fundus as well as the Ezisurg^™^ motorized stapler depending on the availability of the device.

The LDG was performed by only one surgeon (IB), indicated for patients suffering from different esophagogastric benign diseases (Table 1). The techniques adopted were described before^8,9,28,29,30^. During this procedure for gastric transection, mechanic stapler 60–3.5 mms blue cartridge and 45–2.5 mms blue cartridge were used only in a few cases for finishing the transection of gastric fundus. For duodenal transection, gastro-jejunostomy, jejuno-jejunostomy, and jejunal transection, only one stapler was employed, either a mechanic 60–3.5 mms blue cartridge stapler or a motorized stapler, also depending on device availability. Clips were used to stop bleeding of the suture line.

**Figure 1:**
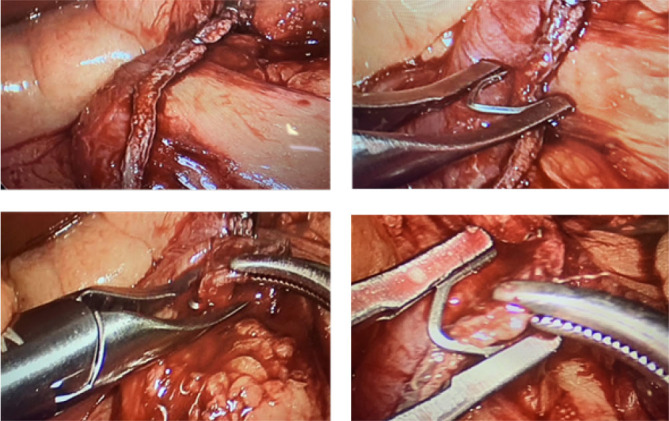
Clips placement for bleeding control of suture line.

The parameters evaluated intraoperatively during surgery were:

a) Total firing time for complete gastric transection;
b) Number of firings to complete the surgical procedure;
c) Number of clips used after complete firing according to the number of firings employed divided by the number of clips employed to obtain complete hemostasis of the suture line (Figure 1);
d) Number of leaks observed after methylene blue intra-gastric installation;
e) Intraoperative difficulties; and
f) Total time of surgical procedure.

After surgery, early outcomes were evaluated for:

a) Early postoperative complications;
b) Total in-hospital stay; and
c) 30-day readmissions.

The use of a mechanic or motorized stapler was chosen depending on the availability of the device at the beginning of the procedure and measurements were established by a nurse during the procedure. Data were recorded in a special protocol designed for this specific study.

Statistical analysis of data was performed with Statistical Package for Social Sciences (SPSS), version 18.0 (IBM Co., Armonk, NY, USA). Chi-square (χ^2^) test was applied where appropriate. A p-value of less than 0.05 was considered significant.

The authors declare that no experiments were performed for this study. All procedures were in accordance with the hospital’s bioethics committee and the 1961 Helsinki Declaration, its later amendments, or comparable ethical standards. To ensure patients’ data confidentiality, the authors adhered to the hospital’s clinical and research protocols for publication. The authors declare that no study patient private data are included in this article and all gave their informed consent before the operation.

## RESULTS

The demographic characteristics of the patients included in this study are very similar in terms of age, sex, and body mass index (Table 1). Comparing the comorbidities in patients operated on for morbid obesity undergoing LSG or LRYGB, there are some differences because in patients with gastroesophageal reflux disease (GERD), LSG is not indicated, and these patients are submitted to LRYGB. The other comorbidities are quite similar in these two groups of patients. On the contrary, in patients undergoing distal gastrectomy, there are other causes such as severe esophagitis with Barrett’s esophagus, conversion to LRYGB after LSG, or the existence of a gastric tumor (Table 2).

**Table 2:** Comorbidities in patients submitted to laparoscopic sleeve gastrectomy, laparoscopic Roux-en-Y gastric bypass, and laparoscopic distal gastrectomy.

Comorbidities	LSG (n=47)	LRYGB (n=30)	LDG (n=21)
Insulin resistance	37	27	-
Diabetes II	2	4	-
HTA	25	10	-
Dyslipidemia	37	27	-
Hypothyroidism	23	4	1
Fat liver	48	30	2
Reflux esophagitis grade C	-	8	6
Primary Barrett´s esophagus	-	3	6
Knee arthrosis	1	2	-
Candy cane and HH post LRYGB	-	-	5
Gastric tumor	-	-	3
Cholelithiasis	1	2	-

Table 3 shows the results obtained in patients submitted to LSG. No significant differences were observed regarding time, number of firings, and number of clips comparing mechanic with motorized stapler. Excessive bleeding that needed suture reinforcement occurred after the use of mechanic stapler, which was associated to more dragged-on surgical procedure. (48±5.9 *vs* 28±2.51 min) (p=0.0001; p<0.05). In patients undergoing LRYGB, during gastric transection and pouch performing, a little difference regarding the total time of firing in favor of the motorized stapler was noted, probably because this latter device has a shorter cartridge. However, a more prolonged time of firing for performing gastrojejunostomy, jejuno-jejuno-anastomosis, jejunal transection, and total time for finishing the procedure was observed in the group using mechanic stapler (p=0.0001; p<0.05) probably due to the waiting time recommended to avoid excessive bleeding of the suture line.

**Table 3:** Intraoperative performance laparoscopic sleeve gastrectomy (n=47).

	Mechanic stapler (n=10)	Motorized stapler (n=37)	p-value
Total time of firing (mean±SD)	3.5±1.89 min	2±0.41 min	<0.0001
Number of firings (mean±SD)	5.8±0.63	5±0.39	<0.0001
Total number of clips (mean±SD)	13±8.16	10±5.39	0.1707
Number clips/each firing (mean)	2.24	1.99	
Number of leaks	-	-	
Intraoperative difficulties	-	-	
Excessive bleeding (n)	2	-	
Necessity of reinforcement (n)	3	-	
Surgical procedure total time (mean±SD)	48±5.9 min	28±2.51 min	<0.0001

SD=standard deviation.

No intraoperative complications were confirmed when comparing the two devices used. (Table 4). Very similar findings were observed in the group of patients undergoing LDG (Table 5). Regarding the postoperative outcome, a prolonged in-hospital stay after mechanic stapler use was identified due to a complication—Clavien-Dindo IIIa (perigastric abscess treated with antibiotics and percutaneous drainage). No other complications and readmissions were reported (Table 6).

**Table 4:** Intraoperative performance laparoscopic gastric bypass (n=30).

	Mechanic stapler (n=14)	Motorized stapler (n=16)	p-value
Gastric transection			
Total time of firing (mean±SD)	2.20±0.10 min	1.66±0.39 min	<0.0001
Number of firings (mean±SD)	3.00±0.00	4.60±0.88	<0.0001
Proximal gastric pouch			
Total number of clips (mean+SD)	3.00±0.96	2.50	0.0459
Number of clips/each firing (n)	0.88	1.84	
Distal gastric remnant			
Total number of clips (mean±SD)	6.30±4.12	4.00±0.0	
Number of clips/each firing (n)	3.50	2.25	0.0333
Number of leaks	-		
Intraoperative difficulties	-		
Excessive bleeding, necessity of reinforcement	-		
Gastrojejunostomy			
Total time of firing (mean±SD)	46.00±4.93 sec	9.17±0.52 sec	p=0.0001
Number of clips	0	0	
Jejuno-jejuno-anastomosis			
Total time of firing (mean±SD)	45.00±11.36 sec	8.96±0.27 sec	p=0.0001
Number of clips	0	0	
Jejunal transection			
Total time of firing (mean±SD)	39.85±5.22 sec	8.06±0.55 sec	p=0.0001
Number of clips (mean±SD)	1.00±0.91	0.80±1.3	p=0.6341
Total time of surgical procedure			
Mean±SD	117.00±19.31min	41.00±2.88 min	p=0.0001

SD: standard deviation; sec: seconds.

**Table 5:** Intraoperative performance laparoscopic distal gastrectomy (n= 21).

	Mechanic stapler (n=13)	Motorized stapler (n=8)	p-value
Duodenal transection			
Total time of firing (mean±SD)	39.00±9.31sec	11.00±1.88 sec	<0.0001
Number of clips (mean±SD)	3.00±1.80	2.00±1.58	p=0.2118
Gastric transection			
Total time of firing	129.00±0.50 sec	33.00±5.86 sec	<0.0001
Number of firings (mean±SD)	3.00±1.16	2.00±0.44	p=0.0317
Total number of clips (mean±SD)	2.00±1.99	1.00±1.30	
Number clips/each firing	0.15±0.55	0	p=0.2232
Number of leaks	-	-	
Intraoperative difficulties	2		
Excessive bleeding	2	0	
Necessity of reinforcement	0		
Gastrojejunostomy			
Total time of firing (mean±SD)	44.00±25.06 sec	13.00±3.39 sec	
Number of clips (mean±SD)	0	0	p=0.0027
Jejuno-jejuno-anastomosis			
Total time of firing (mean±SD)	32.00±20.02 sec	12.00±2.01 sec	
Number of clips (mean±SD)	0	0	p=0.0117
Jejunal transection			
Total time of firing (mean±SD)	26.00±15.34 sec	12.00±0.46 sec	
Number of clips (mean±SD)	0.07±0.27	0	p=0.0194
Total time surgical procedure	185.90±18.50 min	157.30±10.20 min	p=0.0008

SD: standard deviation; sec: seconds.

**Table 6:** Postoperative outcome of patients comparing the type of stapler used for performing the procedure.

	Surgical procedure					
	LSG (n=47)		LRYGB (n=30)		LDG (n=21)	
	Mech	Motor	Mech	Motor	Mech	Motor
	(n=10)	(n=37)	(n=14)	(n=16)	(n=13)	(n=8)
a) Early postoperative complications	-	-	-	-	1*	-
b) Total in-hospital stay	1	1	1	1	3.21^†^	
	**(p=ns)**		**(p=ns)**		**(p=ns)**	
c) 30-day readmissions	0	0	0	0	0	0

LSG: laparoscopic sleeve gastrectomy; LRYGB: laparoscopic Roux-en-Y gastric bypass; LDG: laparoscopic distal gastrectomy; ns: not significant.

*Perigastric abscess (Clavien-Dindo IIIa);

^†^1 patient= 20-day hospital stay.

## DISCUSSION

This study was conducted to compare the early outcome using two different types of staplers in obese patients submitted to LSG, LRYGB, and LDG indicated for Barrett´s esophagus as a primary procedure, redo fundoplication for failed Nissen fundoplication, or conversion to resectional gastric bypass after sleeve gastrectomy^4,9,22,38,43,44^. These procedures are not exempt from postoperative complications including leakage from staple lines, bleeding, and fistula formation^1,13,17,26,27,33,35^. In order to minimize the line suture postoperative complications, the stapling instrument is employed to simplify and optimize the procedure, and facilitate tissue approximation and transection during surgery. These new devices also require less skill from the surgeon. Several different models of motorized staplers (i.e., those for which the staples and knife blade are driven not by manual force but by a power source instead) have been used since 2010. Subsequent versions have been introduced^24,30^. These powered staplers were developed to increase stability and enable more precise stapling relative to non-powered (manual) staplers^42^.

In the literature, few papers focused on the analysis of intraoperative and postoperative outcomes using this type of stapler. Roy et al.^41^ reported the results concerning cost, operative time, and in-hospital stay. In this study, the mean hospital stay was 2.1 days for both the powered and manual stapler groups (p=0.981, p>0.05). Total costs of the hospital, mean supply, and mean operating room were significantly less expensive using the powered stapler (p=0.003, p=0.011, and p=0.009, respectively, p<0.05) The operative time, rate of bleeding and/or transfusions were also significantly lower for the powered stapler group *vs* the manual stapler group. The adjusted rates of 30 (4.4%), 60, and 90-day all-cause readmissions were similar between the groups (all p>0.05)^41^.

Another study evaluated 60 consecutive LSG procedures—30 sleeves using the AEON™ Endostapler in thick mode and 30 using the ECHELON Flex™ Powered Stapler with pulse technique^37^. The authors assessed stapler performance regarding the incidence and degree of staple line bleeding by visualizing bleeding after the final firing. It was analyzed by a third-party blinded evaluator and given a “bleeding score”—a qualitative measure of intra-operative staple-line bleeding (1= no bleeding to 5= profuse bleeding). The AEON™ Endostapler had 15 cases (50%) with no bleeding at the fundus and the ECHELON Flex™ had 7 cases (23%). The authors concluded that AEON™ Endostapler is a significantly drier alternative to the ECHELON Flex™ Powered Stapler, producing a much drier staple line and decreasing the need for other bleeding control methods^37,40^.

Other reports suggested that the AEON™ Endostapler produces a significantly drier staple line, compared to the ECHELON Flex™ Powered Stapler, and is associated with less interventional control of the staple line^37,43,44^.

Rawlins et al.^37^ compared outcomes between the two latest innovations in powered stapling technology—the ECHELON Flex™ GST system (GST) and the SIGNIA™ Stapling System (SIG)—among patients undergoing sleeve gastrectomy for obesity, concerning leak, total hospital costs, length of stay, and operating room time. Then, 30, 60, and 90 days of all-cause inpatient readmissions were also examined. The observed incidence proportion of hemostasis-related complications during surgical admission was lower in the GST group than in the SIG group (0.006% *vs* 0.020%). Differences between the GST and SIG groups were not statistically significant for leakage, total hospital costs, length of stay, operating room time, and all-cause inpatient readmission at 30, 60, and 90 days. GST system has been associated with a lower rate of hemostasis-related complications as compared to SIG. A powered stapler with a GST system has demonstrated safety for use in gastric surgery^11,39^.

The beneficial aspects of the powered device may be derived from:

a) The combination of increased stability, along with superior control of tissue movement with advanced reloads potentially reducing the cause of trauma to tissue;
b) Formation of a more integrated staple line;
c) Speed time of firing;
d) Less effort during surgery; and
e) More favorable cognitive, affective, physiological, and behavioral outcomes^4^.

In our study, the objective parameters were focused on evaluating firing time, bleeding control, leaks after complete firing, and early postoperative complications that have not been published before. The results are similar to other prior studies examining the same selected outcome parameters compared to surgery performed with a non-powered system^1,2,10,20,23,45,46^. In our experience, the main advantage of using a motorized stapler is the total surgical time once finished the procedure. A subjective parameter difficult to evaluate is ergonomic advantages. For us, Ezisurg^TM^ is lighter and easier to use than other motorized devices available in the market.

## CONCLUSIONS

According to the results of this study, the motorized Ezisurg^TM^ stapler offers safety and efficacy as revealed in prior reports, and is relevant since less total time of surgical procedure without intraoperative or postoperative complications were confirmed. However, further controlled prospective studies are needed to confirm the validity of these findings.
